# Can river laws deliver ecological outcomes? Evaluating the Yangtze River protection law through a stakeholder lens

**DOI:** 10.1007/s13280-025-02324-4

**Published:** 2026-02-19

**Authors:** Yunbo Li, Susan Jing Su, Alicia Ying Zhang

**Affiliations:** 1 School of Law, Yangzhou University, 88 South University Avenue, Yangzhou, 225009 Jiangsu China; 2 Faculty of Business and Law, Middlesex University London, The Burroughs, London, NW4 4BT UK; 3 Zhuhai College of Science and Technology, 368 Shengli Road, Tangjiawan, Zhuhai, 519041 Guangdong China; 4 School of Hotel and Tourism Management, The Hong Kong Polytechnic University, 17 Science Museum Road, Hung Hom, Kowloon, Hong Kong SAR China

**Keywords:** Ecological indicators, Legal frameworks, River governance, Stakeholder engagement, Yangtze River Protection

## Abstract

Sustainable river governance increasingly relies on legal frameworks that integrate ecological indicators with coordinated governance mechanisms. This study examines the Yangtze River Protection Law (YRPL), China’s first national legislation for a single river basin, to evaluate how ecological indicators can enhance the law’s capacity to achieve measurable ecological outcomes. Drawing on interviews with 40 stakeholders, including legal practitioners, ecologists, policymakers, and community representatives, this study identifies persistent barriers such as ambiguous goals, the absence of ecological indicators, and uneven enforcement across regions. To address these challenges, the study proposes a multi-dimensional framework that centers on ecological indicators, such as biodiversity recovery and flow stability, while recognizing the enabling role of stakeholder engagement and governance adaptability. The proposed indicators, including biodiversity recovery rates, flow stability, and enforcement consistency, build on existing ecological assessment standards to enhance legal accountability and comparability. By integrating ecological science with legal analysis, the study offers a replicable model for assessing river protection laws and contributes to international debates on how law can operationalize ecological resilience.

**Supplementary Information:**

The online version contains supplementary material available at 10.1007/s13280-025-02324-4.

## Introduction

Rivers are vital lifelines for ecological systems and human civilizations, providing essential ecosystem services such as water supply, biodiversity support, and flood regulation. However, rapid industrialization, urbanization, and climate change have severely disrupted river ecosystems globally (Islam et al. 2024). Challenges such as declining water quality, habitat degradation, and biodiversity loss underscore the urgent need for legal frameworks that can not only regulate human activities but also evaluate ecological outcomes through measurable ecological indicators. This raises a critical research question: Can river protection laws effectively achieve their ecological objectives without such indicators, and what mechanisms are needed to bridge this evaluative gap? We hypothesize that without clearly defined and scientifically validated ecological indicators, and effective cross-regional coordination mechanisms, even well-intentioned river protection laws will struggle to meet their ecological goals.

Legal frameworks play a pivotal role in mitigating threats to river ecosystems by regulating pollution, ensuring sustainable resource use, and preserving ecological integrity. Internationally, policies such as the European Union’s Water Framework Directive (WFD) and the US Clean Water Act (CWA) have been hailed as benchmarks for river protection. The WFD emphasizes achieving “good ecological status” for all water bodies, while the CWA aims to restore and maintain the chemical, physical, and biological integrity of US waters. Despite notable successes, these legal frameworks still face persistent challenges. Observers have pointed out issues including unclear ecological targets, inconsistent enforcement, and limited engagement with the scientific community (Voulvoulis et al. 2017; Keiser and Shapiro 2019). These examples illustrate that even mature legal systems struggle when ecological indicators are vaguely defined or inconsistently applied, making it difficult to translate legal mandates into measurable ecological improvements.

In China, similar gaps are evident. Although river protection legislation has advanced rapidly, ecological outcomes remain uneven: Biodiversity in major basins continues to decline, and water quality improvements are often localized rather than systemic (Liu et al. 2023). For instance, despite the Yangtze River Protection Law (YRPL) being China’s first basin-specific environmental law, its implementation still lacks explicit ecological benchmarks for assessing restoration success and interprovincial coordination efficiency. These patterns suggest that the absence of standardized ecological indicators constrains both accountability and adaptive governance, resulting in symbolic rather than substantive ecological progress.

One critical limitation of many existing river protection laws lies in their inability to incorporate comprehensive and scientifically validated indicators for evaluating ecological outcomes. Indicators such as biodiversity indices, water quality metrics, and hydrological parameters are seldom explicitly integrated into legal mandates, leaving a gap between legislative intent and actual ecological results. For instance, studies have highlighted that the success of policies like the WFD is uneven across member states, largely due to variations in implementation and a lack of clear, measurable ecological benchmarks (Solimini et al. 2008; Hering et al. 2010; Santos et al. 2021). Similarly, in many regions, legal systems focus heavily on compliance with pollutant discharge limits while neglecting broader ecosystem health metrics such as habitat connectivity or species diversity (Ormerod et al. 2010; Harding 2023). This disjunction between legal compliance and ecological recovery underscores a central argument of this study: That law must move beyond procedural control toward performance-based evaluation grounded in ecological indicators.

Although river protection laws exist in many jurisdictions, there remains a striking lack of structured approaches to evaluate their ecological effectiveness. Research often proceeds in disciplinary silos: Legal scholars emphasize institutional and procedural dimensions, while ecologists focus on environmental indicators (Liu et al. 2022, 2023). This fragmentation hampers meaningful evaluation of whether legal frameworks are achieving intended ecological outcomes. Moreover, the role of diverse stakeholders (legal practitioners, environmental scientists, policymakers, and community representatives) has often been neglected (Falkner et al. 2018). Without their perspectives, assessments risk being incomplete and disconnected from practice. For instance, legal practitioners may prioritize enforceability and compliance, while ecologists emphasize long-term ecosystem health, leading to divergent criteria for “success” (Daniel 2024). These differences reflect a deeper institutional gap: Environmental law and ecological science often operate in parallel rather than in partnership, weakening the translation of legal intent into ecological outcomes. Such differences underscore the need for interdisciplinary approaches that integrate ecological science with legal policy in river governance.

This study directly responds to these theoretical and practical gaps by developing an interdisciplinary assessment framework for evaluating the ecological effectiveness of river protection laws, centered on measurable ecological indicators. By synthesizing insights from law, ecology, and policy, it outlines directions for improving ecological outcomes while offering foundational guidance for future research. The framework explicitly incorporates stakeholder perspectives to ensure that proposed indicators are both scientifically valid and institutionally implementable.

The significance of this study lies in highlighting several overlooked dimensions of the current discourse. Many legal frameworks fail to specify measurable ecological indicators, making it difficult to determine whether objectives are achieved in practice. Research approaches remain fragmented, with legal and ecological analyses rarely integrated, producing partial assessments of law’s ecological impacts. Equally, limited stakeholder integration weakens understanding of on-the-ground challenges and undermines efforts to align legal mandates with ecological and social realities. By embedding ecological indicators within a multi-stakeholder evaluative framework, this study bridges the gap between normative legal commitments and empirical ecological performance. As rivers worldwide confront mounting ecological pressures, this study contributes globally relevant insights into how legal frameworks can be evaluated and strengthened. By foregrounding the integration of ecological indicators, enforcement consistency, and participatory governance, it advances the broader effort to harmonize legal design with ecological science in river governance and offers lessons of international relevance.

## Theoretical Framework

Effective river governance sits at the intersection of legal mandates, ecological science, and multi-level stakeholder engagement. The theoretical underpinnings of this study draw from environmental law theory, ecosystem management, and governance models to explain how legal frameworks can be designed and evaluated for ecological effectiveness. At its core, ecological effectiveness refers to the extent to which legal frameworks achieve measurable ecological outcomes through scientifically validated indicators and adaptive enforcement mechanisms (Ormerod et al. 2010; Harding 2023). Achieving this requires moving beyond compliance-based environmental management toward outcome-oriented legal design.

In theory, laws governing rivers should incorporate scientific feedback loops, using ecological indicators to set targets, monitor outcomes, and adjust management actions, thereby aligning legal standards with ecological realities (Wang et al. 2022; Stoffers et al. 2024). However, the translation of ecological science into legal accountability has often been partial and uneven (Voulvoulis et al. 2017). Traditional legal frameworks prioritize command-and-control instruments, which emphasize emission permits and administrative sanctions, while offering limited space for iterative learning. This gap between legal design and ecological complexity highlights a key theoretical challenge: how to institutionalize adaptive mechanisms that allow legal rules to respond to dynamic ecosystems (Rouillard and Spray 2017). In China, this gap is particularly visible. Most environmental statutes rely on administrative targets such as “section-based water quality assessments” that privilege chemical indicators (e.g., COD, ammonia nitrogen) over biological and hydromorphological dimensions of ecosystem health (Barresi 2017; Li and Jin 2023). As a result, legal compliance can be achieved without corresponding ecological recovery, revealing the urgency of developing integrated ecological indicators that can connect law with environmental outcomes.

One important aspect of this theoretical framework is the integration of explicit ecological indicators into legal criteria for success. Building on principles from environmental management, a performance-based legal approach is advocated, wherein metrics such as biodiversity indices, habitat connectivity scores, or minimum ecological flow requirements are embedded in statutory language (Venesjärvi et al. 2015). The EU Water Framework Directive (WFD) represents a paradigmatic example of this institutionalization, codifying “good ecological status” as a legally binding goal (Czúcz et al. 2021). Yet, as Maia (2017) and Hering et al. (2010) note, the WFD’s success has been hindered by inconsistent data systems and local discretion—demonstrating that the existence of indicators does not guarantee enforceability. China’s Yangtze River Protection Law (YRPL) similarly articulates broad ecological objectives but lacks quantifiable benchmarks, leaving courts and agencies without operative standards for judgment or monitoring (Barresi 2017; Wang et al. 2025; Liu et al. 2023). These experiences converge on a theoretical insight: ecological indicators only strengthen law when they are embedded in transparent evaluation systems with clear administrative responsibility and data verification mechanisms.

Governance structure also determines how such indicators are applied. Collaborative and polycentric governance theories argue that complex ecological issues, such as river basin management, require multi-level coordination and the participation of diverse actors (Carr 2015; Galvez et al. 2020). A river basin, often spanning several jurisdictions, exemplifies what governance theorists describe as a “networked system” requiring horizontal and vertical integration (Holt et al. 2012; Opiyo et al. 2022). In China, the Yangtze River Basin Commission and regional ecological compensation agreements attempt to build such coordination, but data sharing and indicator harmonization remain limited, leading to inconsistent interpretations of “ecological restoration success.” As Wu et al. (2024) observe, mismatched objectives between upstream conservation zones and downstream industrial regions generate fragmented enforcement and “paper compliance.” Theoretically, therefore, indicator-based coordination mechanisms, such as basin-wide ecological monitoring councils, are crucial to transform fragmented governance into adaptive, data-driven systems (Guo et al. 2023).

Incorporating diverse expert and local perspectives in legal design is also central to ecological effectiveness. Legal practitioners emphasize enforceability and clarity, while ecologists stress ecosystem resilience; reconciling these views requires sustained collaboration (Daniel 2024). Participatory monitoring and citizen science projects increasingly serve as low-cost methods to validate ecological indicators and enhance transparency (Lim et al. 2022; Soria-Delgado et al. 2025). In China, pilot programs along the Yangtze and Huai Rivers have experimented with “public river chiefs” (民间河长) to supplement official monitoring, reflecting a gradual shift toward inclusive governance (Liu et al. 2020; Guo et al. 2023). These hybrid forms of participation highlight how social legitimacy and scientific validity can reinforce one another. In this study’s theoretical framework, ecological indicators thus function both as normative standards and as social instruments that mediate between scientific knowledge, administrative practice, and community experience.

Taken together, this theoretical synthesis links environmental law, governance theory, and ecological science into a unified evaluative model. It posits that ecological indicators operate as the critical interface between legal intention and ecological outcome, making them indispensable for assessing the real-world performance of river protection laws. By embedding measurable ecological criteria within adaptive and participatory governance systems, laws like the YRPL could evolve from symbolic protection to empirically grounded ecological regulation—a transformation essential for the next phase of China’s environmental governance.

## Materials and Methods

### Case Context: The Yangtze River and Its Legal Framework

The Yangtze River, extending over 6,300 km, is Asia’s longest river and sustains nearly one-third of China’s population. Its basin supports one of the world’s most diverse freshwater ecosystems, including the Yangtze finless porpoise and Chinese sturgeon, while also underpinning agriculture, industry, transportation, and hydropower. Yet decades of industrialization and urbanization have driven severe ecological degradation, water pollution, habitat loss, and biodiversity decline that mirror threats to other iconic rivers such as the Amazon, Ganges, and Nile (Vörösmarty et al. 2010).

In 2021, the Chinese government enacted the Yangtze River Protection Law (YRPL), its first comprehensive legislation dedicated to a single river basin. While river-focused legal frameworks exist elsewhere, such as the Whanganui River legislation in New Zealand, the Columbia River Treaty, and the Mekong Agreement, the YRPL stands out as one of the earliest national statutes passed exclusively for ecological protection and integrated governance at the basin scale. It combines ecosystem health mandates with cross-regional regulatory coordination and introduces a holistic approach to pollution control, biodiversity conservation, and water resource management. In this respect, it represents a milestone in Chinese environmental legislation (Zhou et al. 2025; Wu et al. 2025).

The YRPL has also become a focal point of debate. Advocates view it as a pioneering model for embedding ecological protection into statutory law and aligning China’s governance with global trends in sustainable river management (e.g., comparisons with the EU Water Framework Directive). Critics, however, question whether ambitious legal design can be reconciled with entrenched economic dependence on the river. These controversies highlight why the Yangtze offers a compelling case study: Its ecological and economic significance amplifies the stakes of implementation, while the law’s innovative scope and contested feasibility encapsulate the broader challenges of balancing environmental protection and development in large river basins.

### Study Design

**Fig. 1:**
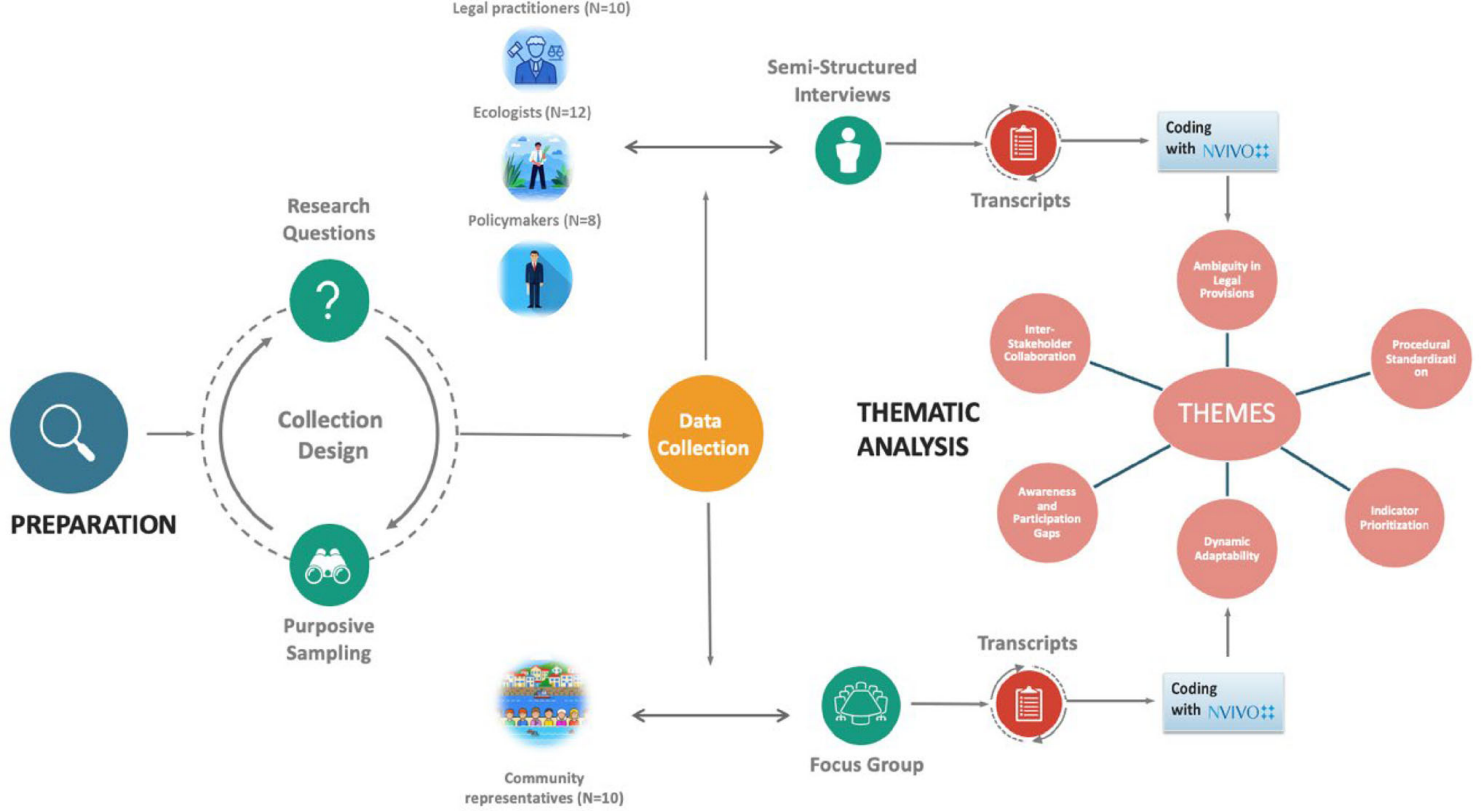
Research flowchart illustrating the qualitative research design, including stakeholder selection, data collection through semi-structured interviews and focus groups, and thematic analysis using NVivo

As shown in Fig. 1, this study adopts a qualitative, exploratory approach to identify the key dimensions necessary for evaluating the ecological effectiveness of river protection laws. This approach was not chosen as a secondary substitute for quantitative tools, but because it offers a helpful lens for capturing the complexity, interpretive dimensions, and evolving practices that underpin environmental governance in real-world contexts. As Flyvbjerg (2006) argues, case-based qualitative research is indispensable when the object of inquiry involves social negotiation, institutional asymmetry, and power-laden implementation processes—as is common in the governance of ecological systems.

In this study, we use the term “stakeholder” to refer to individuals or groups with a legitimate and practical connection to the Yangtze River Protection Law (YRPL), whether through legal design, scientific knowledge, administrative authority, or everyday livelihood. These include legal professionals, ecologists, policy officials, and community members. We are aware of scholarly critiques of the term “stakeholder” for its managerial overtones or exclusionary connotations in some global contexts (Reed et al. 2024). While retaining it here for communicative consistency with legal policy literature, we do so reflexively and remain attentive to the evolving language of participation and justice.

### Participant selection and data collection strategy

The data collection process was structured around two qualitative techniques: semi-structured interviews and focus group discussions. Ethical approval for this study was obtained through the research ethics procedures of Yangzhou University. All participants provided informed consent. All participants were informed in advance of the study’s goals and ethical safeguards. Written consent was secured, and interviews were conducted in strict accordance with protocols ensuring privacy, anonymity, and voluntary participation.

Participants were selected through purposive sampling to ensure diversity across four core actor categories central to the YRPL’s interpretation and implementation:

1. Legal practitioners (e.g., environmental judges, regulatory lawyers);
2. Ecologists (e.g., hydrologists, biodiversity experts);
3. Policymakers (e.g., regional water authorities, local environmental officials);
4. Community representatives (e.g., rural villagers, grassroots NGO leaders, ecotourism entrepreneurs).

A total of 40 participants were interviewed, with their professional affiliations and geographic distribution summarized in Table 1.

**Table 1:** Stakeholder group characteristics and rationale

Stakeholder group	Sample size	Subgroups/professions	Rationale for inclusion	Geographic distribution
Legal practitioners	10	Judges, lawyers, compliance officers, legal advisors	To evaluate legal enforceability, design robustness, and challenges in implementing and interpreting the YRPL	3 from upstream provinces (e.g., Yunnan, Chongqing), 4 from midstream (e.g., Hubei, Jiangxi), 3 from downstream (e.g., Jiangsu, Anhui)
Ecologists	12	Freshwater ecologists, hydrologists, conservationists, climate specialists	To assess ecological indicators, biodiversity concerns, and the integration of scientific thresholds into legal frameworks	4 from research institutes, 5 engaged in applied conservation projects, 3 from academic institutions (e.g., Jiangsu, Hubei, Sichuan)
Policymakers	8	Regional water managers, environmental officials, policy consultants	To understand how policy objectives align with ecological goals, and examine interprovincial coordination challenges	2 from upstream regions (e.g., Sichuan, Chongqing), 3 from midstream (e.g., Hubei, Jiangxi), 3 from downstream (e.g., Anhui, Jiangsu)
Community representatives	10	Fisherfolk, small-scale farmers, NGO leaders, activists, ecotourism entrepreneurs	To explore public awareness, participation, and perceived socioeconomic and ecological impacts of the YRPL	3 from rural communities (e.g., Yunnan, Sichuan), 4 from urban areas (e.g., Shanghai, Jiangsu), 3 from semi-urban regions (e.g., Hubei, Anhui)

Between June and August 2024, semi-structured interviews were conducted with participants from all four groups. To ensure fair and consistent participation, each interview lasted approximately 45–60 min, with only minor variation based on availability. Interviews were conducted in Mandarin Chinese or local dialects, and later translated where appropriate. Interviews followed a flexible guide tailored to each group but anchored in three main domains: (1) perceived strengths and gaps in the YRPL; (2) feasibility and relevance of ecological indicators; and (3) cross-regional and cross-sectoral coordination barriers.

Focus group discussions were conducted separately with community representatives to enhance collective narrative generation and allow for interpersonal dynamics to surface. Each session included 6–8 participants and lasted approximately 90 min. These discussions were moderated by the research team and followed a facilitative, nondirective structure to allow spontaneous themes to emerge, particularly around participation, legitimacy, and regional inequality in ecological protection.

### Data processing and analysis

All interviews and focus groups were audio-recorded with consent and transcribed verbatim. Transcripts were imported into NVivo 14 and analyzed through a multi-step thematic coding process (Wipulanusat et al. 2019; Khoa et al. 2023). Open coding generated initial categories, followed by axial coding to refine links across interview types. To ensure transparency and consistency, the team used memo writing, coding comparison, and NVivo query functions; an independent co-coder reviewed 20% of transcripts. To illustrate how narratives were translated into themes, two anonymized excerpts are presented with codes:

“The law speaks of ‘restoring ecological integrity,’ but without specifying what that means in practice… It’s more of a political slogan than a legally enforceable standard.”

– Environmental law judge, midstream province.

*Coded as: legal ambiguity; lack of measurable targets; symbolic law*

“Most villagers have never heard of the YRPL… People care about the river, but the law feels like it belongs to someone else, far away in the capital.”

– Grassroots NGO coordinator, downstream region.

*Coded as: awareness gap; implementation disconnect; community marginalization*

These excerpts demonstrate how verbatim accounts were coded into themes, highlighting disjunctures between legislative intent and local implementation.

### Validation and limitations

To enhance credibility, a member-checking process was conducted: Preliminary findings and coding summaries were shared with a subset of participants (n = 6) for feedback. Their suggestions led to refinement of several subthemes, especially regarding enforcement disparity and ecological terminology.

A summary of the overall research process is provided in Table 2, including group composition, interview format, and regional balance.

**Table 2:** Data collection overview

Data type	Source	Participant groups	Representative profiles	Purpose
Semi-structured interviews	Legal practitioners	Judges, lawyers, compliance officers, advisors	Senior environmental judge (Wuhan, 25 + years of experience); environmental lawyer (Shanghai, industrial pollution cases); provincial legal advisor (Jiangxi, regulatory audits); compliance auditor (Anhui, private sector focus)	Evaluate legal enforceability, interpret challenges, and analyze gaps in legal text
	Ecologists	Freshwater ecologists, hydrologists, conservationists, climate specialists	Freshwater biologist (Hubei, fish biodiversity focus); hydrologist (Chongqing, flow modeling expertise); wetland ecologist (Jiangsu, riparian habitat restoration); climate change hydrologist (Hubei, resilience of aquatic ecosystems)	Assess ecological indicators, biodiversity recovery, and relevance of hydrological thresholds in legal implementation
	Policymakers	Regional officials, NGO-affiliated consultants, policy planners	Regional water resource manager (Sichuan, interagency coordination); provincial environmental director (Jiangxi, enforcement oversight); policy consultant (Shanghai, NGO collaboration focus)	Examine policy alignment with ecological goals, interprovincial coordination, and resource allocation strategies
Focus groups	Community representatives	Rural, urban, and semi-urban stakeholders	Fisherfolk (Yunnan, traditional livelihoods); small-scale farmer (Sichuan, floodplain agriculture); urban NGO leader (Jiangsu, advocacy and outreach); riverfront ecotourism Entrepreneur (Anhui); youth environmental activist (Jiangsu)	Explore public awareness, grassroots engagement, and socioeconomic impacts of the YRPL on diverse communities

While this study does not aim for statistical generalizability, its analytical transferability lies in revealing how institutional complexity and social engagement shape the effectiveness of river protection laws. Future research may adopt a mixed-methods approach, incorporating ecological monitoring data or large-scale perception surveys, to triangulate findings and broaden scope. However, such quantitative extensions should build upon, rather than replace, the interpretive richness provided by in-depth qualitative analysis.

This methodology foregrounds the interpretive, negotiated, and often contested nature of law in practice. It provides a grounded, stakeholder-informed understanding of how ecological legal instruments operate not only on paper, but in the lived realities of courts, planning bureaus, villages, and riverbanks.

## Results and discussion

### Legal framework gaps: The Achilles’ heel of implementation

The findings reveal significant shortcomings in the clarity and operational structure of the Yangtze River Protection Law (YRPL), highlighting how conceptual ambiguity and institutional fragmentation weaken its ecological effectiveness. As shown in Appendix Table S1, the code Legal Ambiguity emerged as a persistent theme among legal practitioners and ecologists alike. Participants repeatedly pointed out that core legal phrases such as "restoring ecological integrity" or "promoting green transformation" remain too abstract to serve as the basis for legal enforcement or ecological evaluation. In short, the statute articulates aspirational aims without specifying the ecological indicators by which those aims would be judged.

One senior environmental judge noted bluntly:

“We cannot enforce a value statement. ‘Restoring ecological integrity’ sounds good, but what exactly are we asking of a developer, or a county government? What level of wetland restoration? What species count? If there's no quantifiable target, then in court it becomes unenforceable. It's philosophy, not law.”

– Environmental judge, midstream province.

Legal compliance officers raised similar concerns, particularly about the growing gap between strategic ambition and operational instruments:

“There’s a lot of symbolic ambition in the YRPL, but very few operational definitions. As local compliance officials, we often struggle to explain to companies or county departments what precisely they need to do. Should they plant trees, reduce effluent by 10%, or create habitat corridors? The law doesn't say.”

– Legal compliance officer, downstream province.

The ambiguity surrounding key ecological objectives not only hinders litigation and administrative enforcement, but also creates uncertainty for regulated actors, local governments, and NGOs seeking to align their actions with the law. Critically, this is a problem of *standards*, not merely of effort: Without named and validated ecological indicators (e.g., biodiversity baselines, environmental flow thresholds, or habitat connectivity ratios), discretion expands while accountability shrinks.

Beyond the abstractness of its goals, the YRPL also suffers from implementation inconsistencies (previously “procedural inconsistencies”), particularly in how it is applied across provincial jurisdictions. Two distinct categories emerged from interviews and coding: (i) *ambiguous provisions*: text too vague to be operationalized; and (ii) *enforceable provisions applied unevenly*: clear rules that exist but are implemented with varying intensity or method. Table 3 highlights these disparities as a major bottleneck for effective national coordination. Participants frequently described the current enforcement environment as fragmented and uneven.

**Table 3:** Themes and subthemes extracted from stakeholder interviews (*Source*: Authors’ own compilation based on stakeholder interviews and thematic coding using NVivo 14)

Theme	Subtheme	Detailed description	Rationale	Key stakeholder insight
Legal clarity and design	Ambiguity in legal provisions	Core clauses (e.g., “restore ecological integrity”) lack named, validated ecological indicators (biodiversity baselines, environmental flow thresholds, connectivity ratios), making them difficult to operationalize in litigation or inspections	Ambiguity expands discretion and shrinks accountability; evaluation cannot proceed without explicit standards	“We cannot enforce a value statement… without a quantifiable target, it becomes unenforceable”—Environmental judge, midstream
	Implementation inconsistencies	Two coexisting patterns: capacity-based (limited personnel/tech, upstream) and coordination-based (conflicting mandates, cross-provincial misalignment, downstream)	Disparate practices undermine uniform compliance and data comparability across provinces	“Same law, different playbook… inconsistency begins when each bureau interprets it its own way.”—Planner, downstream
Ecological integration	Indicator scope and validation	Clarifies boundary between ecological condition indicators (biological/chemical/hydromorphological, e.g., biodiversity recovery, WQI, flow stability, connectivity, sediment/morphodynamics) and governance/enforcement indicators (e.g., application rate, data sharing). Anchors to GB3838-2002 and WFD dimensions; requires baselines, frequency, and spatial resolution (reach level)	Avoids indicator conflation; ensures indicators are measurable, comparable, and legally serviceable	“Start with what you can measure, then improve precision—absence of indicators means absence of law in practice.”—Ecologist
	Dynamic adaptability	Five-year indicator reviews; pre-specified uncertainty treatment; seasonal sampling cadence; reach-level reporting; sentinel species lists updatable with new evidence	Aligns law with ecosystem dynamism; creates a legal feedback loop from monitoring to revision	“A living river needs living indicators; the system should evolve with the river.”—Ecologist
Public engagement	Awareness and participation gaps	Low awareness, episodic activities; activity counts (meetings, campaigns) often misreported as ecological achievements	Participation is necessary but not sufficient; it is an enabler, not evidence of ecological condition	“We’re counting actions, not outcomes.”—NGO coordinator, lower Yangtze
	Interstakeholder collaboration	Engagement as enabler via three mechanisms: co-producing data (fills spatiotemporal gaps), contextualizing baselines, supporting periodic review (flag anomalies). Requires basin-wide data sharing protocols and joint validation workshops	Converts engagement into verifiable data flows that support indicator use and review; reduces “ghost provisions.”	“When weekly citizen data triggers inspections, provisions are more likely to be used.”—Environmental lawyer
Governance and enforcement	Cross-provincial coordination	Lack of unified inspection protocols, reporting templates, and shared funding pools; data non-recognition across borders	Harmonization ensures comparability and trust; precondition for uniform enforcement	“We submitted records; the next province wouldn’t recognise the form.”—Volunteer, midstream
	From benchmarks to use	Benchmarks do not equal enforceability; requires indicator application metrics (e.g., YRPL Application Rate), trigger thresholds for inspections, and transparent auditing	Tracks how often indicator-based provisions are invoked; turns numbers from symbolic to actionable	“Quantification without application creates an illusion of success.”—Environmental lawyer

One regional water bureau director reflected:

“There’s no standardized protocol across provinces. In some places, they use satellite monitoring to track land use change. In others, inspections are still done with handwritten forms. The result is inconsistent data, enforcement delays, and frustration at the grassroots level.”

An ecologist working with a national NGO expressed concern about the consequences for scientific cooperation:

“We tried to run a cross-provincial study on fish biodiversity, but one region refused to share monitoring data, saying it wasn’t legally required. Another said they hadn’t collected any. This legal fragmentation is making scientific integration and accountability extremely difficult.”

Such accounts echo international experiences, such as those under the EU’s Water Framework Directive, where variable local interpretations have been shown to dilute the directive’s overall impact (Hering et al. 2010). But an additional mechanism surfaced strongly in this study: *ghost provisions*, rules that exist on paper yet are rarely invoked in inspections, administrative penalties, or court decisions. Quantifiable benchmarks alone do not guarantee use; what matters is whether provisions are routinely applied and reviewable.

To address these legal and implementation weaknesses, Tables 3 and 4 propose the integration of specificity, enhancing mechanisms within the YRPL. These include mandatory ecological benchmarks, such as measurable pollutant discharge limits, minimum water flow thresholds, or habitat restoration targets, as well as adaptive clauses that allow the legal framework to evolve in step with emerging environmental data. Equally important, the framework should couple benchmarks with enforceability metrics. For example, a “YRPL Application Rate” (number of judicial or administrative cases explicitly citing ecological indicator provisions per year), inspection frequency tied to indicator exceedances, and interprovincial data sharing obligations aligned with existing national standards (e.g., surface water quality classifications) to reduce discretion drift. Embedding both *what to measure* (ecological indicators) and *how often it is applied* (usage-based enforcement indicators) can shift the YRPL from rhetorical aspiration toward measurable, reviewable, and thus enforceable ecological governance.

**Table 4:** Proposed indicators for evaluating the Yangtze River Protection Law (*Source*: Authors’ own compilation based on stakeholder interviews and thematic coding using NVivo 14)

Dimension	Proposed indicator	Operational definition/measurement logic	Primary data source	Example of application
Ecological indicators	Biodiversity recovery index	Annual change in sentinel species populations (e.g., Yangtze finless porpoise, Chinese sturgeon) benchmarked to documented baselines; includes confidence interval and five-year review for trend validation	Longitudinal biodiversity surveys; academic and NGO datasets	Evaluate habitat restoration effectiveness and species recovery progress across provinces
	Hydrological flow stability	Percentage of river reaches meeting minimum environmental flow thresholds per season with exception rules for drought events	River basin hydrological reports; dam operation records	Assess compliance of upstream dam operations with ecological flow targets; trigger adaptive water release policies
	Water quality index (WQI)	Composite index of TN, TP, COD, DO and NH_3_-N aligned with China’s *Surface Water Environmental Quality Standard* (GB 3838–2002). Calculated monthly at reach scale	Provincial environmental monitoring stations	Compare interprovincial WQI trends and link results to funding allocation for restoration projects
	Habitat connectivity ratio	Longitudinal passability of migratory routes and riparian continuity derived from remote sensing and fishway functionality audits	Remote sensing imagery; hydroecological field surveys	Track corridor reconnection and fish migration success after barrier removal or dam retrofitting
	Sediment and morphodynamic stability	Deviation of sediment load and bank erosion rate from reference conditions; reviewed every five years	Hydrogeomorphological monitoring networks	Detect morphological alterations linked to infrastructure and inform channel rehabilitation targets
Governance indicators	YRPL Application Rate	Number of administrative or judicial cases per year explicitly invoking YRPL ecological indicator provisions; serves as usage-based enforcement metric	Court decisions; agency penalty records	Gauge how often indicator-based provisions are applied in practice; identify “ghost provisions.”
	Cross-provincial data sharing index	Percentage of provinces submitting standardized indicator datasets to the basin authority on schedule	Basin data exchange platform; annual progress reports	Measure coordination and information flow across jurisdictions; highlight data gaps for targeted support
	Regional enforcement uniformity index	Statistical variation (coefficient of variation) in inspection frequency, compliance rates, and penalties across provinces	Provincial enforcement reports; central audits	Identify systematic regional inequalities and inform redistribution of resources and oversight capacity
	Interdisciplinary integration mechanism	Existence of joint indicator review committees and annual science–policy dialogues; records of updated baselines and method revisions	Basin management authority minutes; committee reports	Track progress of knowledge co-production and adaptive review (see “Interdisciplinary integration: the path forward” section)

### Ecological indicators: Bridging science and policy

The interviews revealed that most stakeholders regarded the absence of clearly defined ecological indicators as the single most significant weakness of the YRPL. Ecologists, policy officials, and legal practitioners all emphasized that without measurable reference points, the law’s commitment to “restoring ecological integrity” remains rhetorical. As one senior hydrologist remarked, “We have slogans, not standards. Everyone agrees on protection, but no one agrees on what success looks like.”

Across interviews, participants repeatedly described a structural tension between what should be measured and what can realistically be measured. Ecologists tended to view ecological effectiveness through scientific variables: biodiversity recovery, hydrological stability, habitat connectivity, and nutrient load reduction, while administrators preferred indicators that could be standardized and reported through existing monitoring systems. A provincial compliance officer explained: “We measure pH and ammonia nitrogen because that’s what the inspection forms require. No one asks for fish migration or sediment balance.” Such comments reveal an institutional bias toward administratively convenient data rather than ecologically meaningful metrics.

Several respondents also questioned the confusion between ecological and governance indicators. A policy consultant noted that, “When reports list the number of coordination meetings or public campaigns as ecological achievements, the concept itself becomes diluted.” In practice, indicators relating to participation or enforcement are essential for implementation but cannot substitute for direct ecological measurement. This distinction between ecological condition indicators and social or governance indicators emerged as a recurring theme (see Table 3), clarifying why evaluation remains inconsistent across provinces.

Respondents further emphasized that the lack of operational definitions undermines accountability. Without numerical thresholds or baseline data, enforcement agencies hesitate to sanction violations, and courts avoid quantitative judgments. As an environmental lawyer explained, “If you can’t prove deviation from a standard, you can’t prove damage.” This logic was echoed by ecologists who argued that even imperfect ecological metrics would be better than purely symbolic clauses. “Start with what you can measure,” one researcher said, “then improve precision later, but absence of indicators means absence of law in practice.”

To address these concerns, the study synthesized stakeholder suggestions into a set of representative ecological indicators, summarized in Table 4 that integrate scientific measurability with legal usability. These include parameters such as biodiversity recovery, water quality, flow stability, and habitat connectivity. Importantly, interviewees agreed that any indicator system must be linked to baseline data, spatial consistency, and periodic review, rather than fixed numerical targets detached from ecological context. An ecologist summarized this view: “A living river needs living indicators. The system should evolve with the river, not freeze it in time.”

Overall, the qualitative evidence highlights that the YRPL’s weakness is not only the lack of data but the lack of a feedback mechanism connecting ecological monitoring to legal enforcement. As one midstream policymaker concluded, “Indicators are not numbers, they are conversations between science and law.” Embedding such conversations into statutory review processes, through adaptive indicator frameworks, would transform ecological measurement from a bureaucratic exercise into a genuine tool of legal accountability.

### Stakeholder engagement: A missing catalyst for success

Stakeholder engagement emerged as a central yet fragile element of the YRPL’s implementation. Interviewees repeatedly cautioned against conflating governance activity with ecological achievement. An NGO coordinator working along the lower Yangtze explained at length:

“We have had plenty of campaigns and conferences. Officials like to list how many meetings were held, how many volunteers joined clean-ups, or how many posters were distributed. But when I ask what the river gained from all this, nobody can show me a single figure on fish stocks or wetland recovery. We are counting actions, not outcomes.”

Her frustration captures a wider sentiment that participation indicators, such as number of events, attendance, media coverage, measure social mobilization, not ecological condition. As several practitioners noted, such metrics are vital for coordination and awareness but cannot stand in for ecological indicators that describe the state of the ecosystem (see Table 3).

At the same time, many respondents viewed engagement as an enabling mechanism rather than a substitute for scientific measurement. A community hydrologist from Hubei elaborated:

“Our group takes weekly samples from a small tributary. It’s not part of the official network, but when we notice a sudden drop in dissolved oxygen, we alert the township bureau. Sometimes they find a nearby discharge. If these data were linked to the basin database, they could trigger real enforcement.”

This narrative illustrates how community monitoring and citizen science initiatives can fill spatial and temporal gaps left by formal observation networks. Through such co-production of data, engagement helps generate the evidence base that ecological indicators depend on. Several interviewees emphasized that engagement becomes meaningful only when it creates a feedback loop: local observations feeding into provincial databases and, in turn, informing enforcement or indicator review. As one environmental lawyer noted, “Without verifiable local data, it is hard for a court to prove ecological harm. Engagement gives us the facts we can stand on.”

Stakeholders also linked participation to consistency and comparability across provinces. A midstream volunteer reflected on the frustration caused by incompatible data formats:

“We submit our bird-count records to the provincial office, but when we share them with the next province downstream, they say the form is different and they can’t use it. It feels like the river ends at the border.”

Such accounts reveal that fragmented data systems undermine both participation and indicator standardization. Participants therefore called for basin-wide data sharing protocols and joint validation workshops, where citizen groups and officials could review results together. As summarized in Table 4, engagement is most effective when it supports data sharing, verification, and cross-provincial coordination, not when it merely multiplies meetings.

Overall, the evidence portrays engagement as a catalyst rather than a proxy for ecological effectiveness. It creates the social and informational infrastructure through which ecological indicators can be operationalized, audited, and adapted over time. As a regional policy officer concluded,

“Indicators are the language of science; participation is how that language gets translated into daily governance. When people measure the river, they start protecting it, and when agencies listen, the law becomes real.”

### Enforcement disparities: Addressing regional inequalities

The enforcement of the YRPL remains highly uneven across the Yangtze River Basin, reflecting both economic and institutional asymmetries. Policymakers frequently pointed to resource gaps between economically developed downstream provinces and less developed upstream regions, a recurring theme categorized under Implementation Inconsistencies in Appendix Table S1. For many upstream authorities, enforcement is constrained not by a lack of will, but by material capacity. A municipal officer from Sichuan described the situation vividly:

“Our office has only three inspectors responsible for nearly two hundred kilometres of river. We do not have drones, real-time sensors, or access to ecological modelling. Most of our budget goes to routine waste management. The law expects comprehensive monitoring, but it never specifies who pays or how the data should be collected. So we improvise, and most of the time we are just reporting instead of inspecting.”

By contrast, officers in wealthier provinces encountered a different problem, namely policy overload and regulatory contradiction rather than scarcity. An environmental planner from Jiangsu explained:

“We are told to prioritise green development but also to increase industrial output. In the same week I was approving a logistics hub and writing a wetland-restoration plan. These directives come from different departments with no clear hierarchy. When two ‘green’ policies conflict, each bureau interprets the law its own way. That is where inconsistency begins.”

Such accounts reveal that regional disparity arises from two coexisting forms of inconsistency: *capacity-based* (limited personnel and technology) and *coordination-based* (conflicting mandates and fragmented oversight). Together, they create a mosaic of enforcement styles that undermines the YRPL’s credibility as a basin-wide statute. A legal practitioner in Hubei summarized the consequence:

“Whether the law is applied depends less on the statute itself and more on the province’s priorities and the governor’s attitude. That is not rule of law, that is rule of discretion.”

These interviews confirm that the problem is not simply one of fiscal inequality but of missing interjurisdictional accountability. Without unified inspection protocols, common reporting templates, and shared funding pools, the YRPL’s enforcement remains contingent and localized. Even where provinces have introduced quantifiable benchmarks, such as emission limits or restoration targets, these provisions often function as ghost provisions, existing in legal text but rarely invoked in administrative penalties or litigation. As one environmental lawyer observed:

“Indicators look convincing on paper, but they do not compel action unless they are tied to review or sanction. Some provinces report 95 percent compliance because they never open a case. Quantification without application simply creates an illusion of success.”

International comparison highlights what is currently missing. Under the US Clean Water Act, federal agencies retain baseline authority to enforce water quality standards and allocate technical and financial assistance to states according to verified performance data. Judicial oversight and public disclosure ensure that numeric targets are actionable rather than symbolic (Keiser and Shapiro 2019). The YRPL, by contrast, decentralizes implementation without providing equivalent vertical accountability or transparent data auditing. As one policymaker reflected, “Our coordination meetings are frequent, but no one checks whether each province measures the same thing or reports it the same way.”

To mitigate these disparities, Table 4 proposes the Regional Enforcement Uniformity Index, designed to monitor variations in inspection frequency, compliance rates, and penalties issued across provinces. Such an index would allow the central authority to redistribute oversight resources and set minimum enforcement baselines, while also revealing where institutional arrangements systematically disadvantage certain regions. More importantly, combining this index with indicator application metrics, which track how often ecological provisions are used in administrative or judicial processes, could convert the YRPL from a patchwork of good intentions into a genuinely enforceable framework.

### Interdisciplinary integration: The path forward

The findings underscore that interdisciplinary integration is not an aspirational ideal but a structural necessity for the YRPL’s success. As ecological challenges become more systemic and multi-dimensional, river protection laws can no longer be designed or implemented within disciplinary silos. The subtheme dynamic adaptability in Table 3 highlights the need for sustained collaboration among scientists, legal experts, and policymakers to ensure that governance remains responsive to evolving socio-ecological conditions. Yet the interviews make clear that integration remains fragile and uneven.

A senior hydrologist from a midstream research institute reflected on this disconnect in detail:

“Our research reports are often published two years after the law or regulation has already been issued. By the time our data are available, the legal text is fixed. When enforcement begins, we find that the targets don’t fit the ecological reality. For example, sediment budgets and fish-migration models can show where restoration should happen, but the legal mandate doesn’t mention them. We end up managing a moving river with static law.”

From the policy side, a former advisor described the same divide from within the bureaucracy:

“Lawmakers are uncomfortable with uncertainty. They want a number that can be regulated: a flow, a pollutant level, a species count. But ecosystems don’t obey fixed thresholds. Without legal flexibility and scientific literacy inside drafting teams, we risk producing elegant but empty rules.”

These reflections reveal that the problem is institutional rather than motivational: the absence of mechanisms that systematically translate ecological knowledge into legal language, and legal mandates back into scientific research questions. Participants repeatedly suggested creating joint indicator review committees and annual science and policy dialogues at the basin level to bridge this gap (see Table 4). Such bodies could coordinate data standards, interpret new findings, and recommend updates to indicator baselines every five years, thereby linking law to living science.

The YRPL provides a unique opportunity to model such integration in practice. By embedding scientifically derived indicators (such as the *Biodiversity Recovery Index* and *Hydrological Flow Stability*) and coupling them with legally mandated interdisciplinary reviews, the law could evolve from a symbolic framework into a learning system. A downstream policymaker summarized this ambition:

“What we need is not another campaign, but a place where judges, ecologists, and engineers sit at the same table. Only then can the law breathe with the river.”

More broadly, these insights suggest a shift toward what scholars term reflexive legal ecology, a governance model in which knowledge co-production is institutionalized rather than improvised, and where law continuously learns from ecological feedback (Voulvoulis et al. 2017; Rouillard and Spray 2017). In this sense, interdisciplinary integration becomes the condition for both scientific credibility and legal legitimacy. For China, establishing such iterative, multi-actor processes could transform the YRPL into a benchmark for adaptive and participatory river governance, with relevance far beyond the Yangtze Basin.

## Conclusion

This study develops an interdisciplinary, stakeholder-informed framework for evaluating the ecological effectiveness and implementation consistency of river protection law, using the Yangtze River Protection Law (YRPL) as its empirical focus. Through interviews with legal practitioners, ecologists, policymakers, and community representatives, five interrelated dimensions emerged as decisive for translating legislation into measurable ecological outcomes: legal clarity, ecological indicators, stakeholder engagement, implementation consistency, and interdisciplinary integration. Together, they reveal that ecological law is only as strong as the institutions and data systems that sustain it.

A central contribution of this research lies in demonstrating how measurable ecological indicators can bridge law and science without reducing ecology to numbers. Indicators such as biodiversity recovery, hydrological stability, water quality indices, and habitat connectivity provide legal benchmarks that are scientifically verifiable. Yet, as participants repeatedly stressed, quantification alone does not guarantee enforcement. Without mechanisms that ensure the routine use of these indicators, such as application tracking, cross-provincial data sharing, and adaptive review, legal benchmarks risk becoming “ghost provisions”. Hence, ecological effectiveness depends not merely on measurement but on the institutional circulation of data, responsibility, and sanction.

Equally important is the empirical insight that public engagement functions as an enabler of indicators rather than a proxy for ecological success. Citizen monitoring, community reporting, and basin-wide data sharing protocols help transform participation from symbolic activity into an operational feedback system. In this way, engagement supplies the informational infrastructure that allows ecological indicators to be reviewed, verified, and locally grounded, strengthening both compliance and legitimacy.

From a theoretical standpoint, the study advances debates on adaptive governance and reflexive legal ecology. It proposes that environmental law should operate as a learning system, one that continuously incorporates new ecological evidence, interdisciplinary collaboration, and social feedback. This approach moves beyond static compliance models toward co-produced governance, where scientists, judges, and citizens collectively define and revise what ecological integrity means in practice. In this sense, the YRPL is not only a case study of China’s evolving environmental governance but also a test of how law can learn from ecology without losing its normative force.

Methodologically, the study’s qualitative scope offers depth rather than breadth. Its purposive sample highlights key stakeholder perspectives but cannot capture the full diversity of basin experiences. Nevertheless, the insights generated here provide a foundation for subsequent empirical testing of the proposed indicators and enforcement metrics. Future research should combine longitudinal ecological data with legal case analysis to assess how indicators perform across jurisdictions and how interdisciplinary review mechanisms can be institutionalized in other contexts.

More broadly, the YRPL illustrates both the promise and the fragility of legal environmentalism in a complex socio-ecological system. It demonstrates that ambitious legislation can catalyze new forms of coordination and learning, but also that implementation gaps, uneven capacity, and the inertia of disciplinary boundaries remain formidable obstacles. Bridging these divides requires not only better indicators but a legal culture capable of listening to rivers as dynamic systems—a reflexive, adaptive practice that turns law from a static text into a living process of ecological accountability.

## Supplementary Information

Below is the link to the electronic supplementary material.

Supplementary file1 (PDF 563 KB)

## Data Availability

The interview and workshop records data are private due to confidentiality agreements and ethical considerations. Access to the data is restricted to ensure participant anonymity and compliance with institutional ethical guidelines. Any requests for data access will be considered on a case-by-case basis, subject to appropriate approvals.
