# Supplementary material for: Can river laws deliver ecological outcomes? Evaluating the Yangtze River protection law through a stakeholder lens
Source: Ambio. 2026 Feb 19;55(8):1918–32. doi: 10.1007/s13280-025-02324-4 (PMC13319271; doi:10.1007/s13280-025-02324-4)
Supplement: Supplementary file 1 — Supplementary file1 (PDF 563 KB) [file 13280_2025_2324_MOESM1_ESM.pdf]

Electronic Supplementary Material (ESM)
Supplementary file S1

Journal: AMBIO
Article number: 2324
DOI: 10.1007/s13280-025-02324-4

Article title:
Can river laws deliver ecological outcomes? Evaluating the Yangtze River protection law through a stakeholder lens

Authors:
Yunbo Li
Susan Jing Su
Alicia Ying Zhang

Corresponding author:
Susan Jing Su
Email: js2371@live.mdx.ac.uk

Contents of this supplementary file:
Table S1. Coding Framework for Thematic Analysis

Appendix

Table S1: Coding Framework for Thematic Analysis

Primary Code	Definition	Secondary Code	Description	Illustrative Quotation	Frequency	Stakeholder Group
Legal Framework Gaps	Challenges arising from vague or incomplete legal provisions that reduce the YRPL's ecological enforceability.	Ambiguity in Goals	Unclear ecological objectives and enforcement priorities; legal terms such as "restoring ecological integrity" lack measurable standards.	"The statute refers to 'restoring ecological integrity,' but the absence of quantifiable benchmarks renders this a rhetorical aspiration rather than an actionable mandate."	19	Legal Practitioners, Ecologists
		Implementation Inconsistencies	Variation in interpretation and enforcement across jurisdictions; includes capacity-based (limited resources, technology) and coordination-based (conflicting mandates) inconsistencies.	"The lack of procedural uniformity across provinces creates a patchwork regulatory system where ecological standards fluctuate arbitrarily."	14	Policymakers
		Ghost Provisions	Statutory clauses or quantified benchmarks that exist on paper but are rarely applied or cited in inspections, administrative penalties, or court decisions.	"Indicators look convincing on paper, but they don't compel action unless they are tied to review or sanction."	7	Legal Practitioners
Ecological Integration	The degree to which ecological science and indicators are embedded within legal frameworks and enforcement mechanisms.	Indicator Scope & Validation	Absence or narrow use of ecological indicators (biodiversity, flow, water quality, connectivity, sediment metrics); insufficient reference to national (GB 3838-2002) or international (WFD) standards; limited baseline and review mechanisms.	"A law without explicit ecological thresholds is like a contract without performance clauses—practically unenforceable."	28	Ecologists
		Biodiversity Complexity	Difficulty of representing ecosystem diversity and trophic interactions within legal definitions.	"Biodiversity isn't just about counting species; it involves interdependent habitats the law overlooks."	12	Ecologists
Stakeholder Engagement	The role and quality of stakeholder involvement in monitoring, implementation,	Public Awareness & Participation Gaps	Low awareness and limited engagement; activities such as meetings or campaigns are often reported as ecological	"We have many meetings and posters, but no one can show fish recovery data—we're counting	17	Community Representatives

Primary Code	Definition	Secondary Code	Description	Illustrative Quotation	Frequency	Stakeholder Group
	and ecological learning.		outcomes, blurring governance and ecological indicators.	actions, not outcomes."		
		Participation and Data Co-production	Community monitoring and citizen science provide data to fill temporal and spatial gaps; engagement meaningful only when it produces verifiable data that inform indicator review.	"Our volunteers check fishway passability weekly; that tells a different story than an annual audit."	10	NGO Leaders, Volunteers
		Cross-Provincial Collaboration	Inconsistent data formats and reporting standards across provinces hinder cooperation and indicator comparability.	"We submitted bird-count records, but another province refused to recognise them."	8	NGOs, Volunteers
Governance & Enforcement	Institutional arrangements and administrative performance influencing ecological law implementation.	Regional Enforcement Disparities	Enforcement capacity uneven across regions; upstream scarcity versus downstream regulatory overload.	"Our office has three inspectors for two hundred kilometres of river… we improvise instead of inspecting."	11	Policymakers, Inspectors
		From Benchmarks to Use	Tracking how often ecological provisions are actually invoked through enforcement metrics (e.g., YRPL Application Rate, case frequency).	"Quantification without application creates an illusion of success."	9	Legal Practitioners
Interdisciplinary Integration	The interaction between scientific, legal, and policy communities in the law's design, monitoring, and review.	Dynamic Adaptability	Five-year indicator reviews; integration of new ecological data; adaptive management; recognition of uncertainty and evolving baselines.	"A living river needs living indicators—the system should evolve with the river."	8	Ecologists, Policymakers
		Science–Policy Translation Mechanisms	Creation of joint indicator-review committees and annual science–policy dialogues to institutionalise interdisciplinary coordination.	"What we need is a place where judges, ecologists, and engineers sit at the same table—only then can the law breathe with the river."	6	Policymakers, Scientists

Source: Authors' own compilation based on stakeholder interviews and thematic coding using NVivo 14.
